# The Alternative Role of Enterobactin as an Oxidative Stress Protector Allows *Escherichia coli* Colony Development

**DOI:** 10.1371/journal.pone.0084734

**Published:** 2014-01-02

**Authors:** Conrado Adler, Natalia S. Corbalan, Daiana R. Peralta, María Fernanda Pomares, Ricardo E. de Cristóbal, Paula A. Vincent

**Affiliations:** Departamento de Bioquímica de la Nutrición, INSIBIO (Consejo Nacional de Investigaciones Científicas y Técnicas-Universidad Nacional de Tucumán) San Miguel de Tucumán, Tucumán, Argentina; University of Massachusetts Medical School, United States of America

## Abstract

Numerous bacteria have evolved different iron uptake systems with the ability to make use of their own and heterologous siderophores. However, there is growing evidence attributing alternative roles for siderophores that might explain the potential adaptive advantages of microorganisms having multiple siderophore systems. In this work, we show the requirement of the siderophore enterobactin for *Escherichia coli* colony development in minimal media. We observed that a strain impaired in enterobactin production (*entE* mutant) was unable to form colonies on M9 agar medium meanwhile its growth was normal on LB agar medium. Given that, neither iron nor citrate supplementation restored colony growth, the role of enterobactin as an iron uptake-facilitator would not explain its requirement for colony development. The absence of colony development was reverted either by addition of enterobactin, the reducing agent ascorbic acid or by incubating in anaerobic culture conditions with no additives. Then, we associated the enterobactin requirement for colony development with its ability to reduce oxidative stress, which we found to be higher in media where the colony development was impaired (M9) compared with media where the strain was able to form colonies (LB). Since *oxyR* and *soxS* mutants (two major stress response regulators) formed colonies in M9 agar medium, we hypothesize that enterobactin could be an important piece in the oxidative stress response repertoire, particularly required in the context of colony formation. In addition, we show that enterobactin has to be hydrolyzed after reaching the cell cytoplasm in order to enable colony development. By favoring iron release, hydrolysis of the enterobactin-iron complex, not only would assure covering iron needs, but would also provide the cell with a molecule with exposed hydroxyl groups (hydrolyzed enterobactin). This molecule would be able to scavenge radicals and therefore reduce oxidative stress.

## Introduction

Iron is essential for every organism, and bacteria are not an exception. Iron is present in bacterial proteins as [Fe S] clusters, and in heme groups [1]. Under aerobic conditions and at physiological pH, iron is present in the Fe^+3^ state and forms insoluble hydroxides and oxyhydroxide precipitates [2]. To acquire iron, bacteria have developed sophisticated strategies involving the production of iron chelators termed siderophores. Once secreted to the environment, siderophores bind iron with high affinity and import it into the bacterial cytoplasm via specific membrane receptors [1]–[3]. In nature, there is a myriad of siderophores and they all belong to a few structural classes, including catecholate, carboxylate, hydroxamate, and mixed ligand siderophores [4]. While it is considered that all siderophores play an equivalent role, in terms of iron uptake, their structural variety suggests functional differences.

*Escherichia coli* synthesizes the catechol siderophore, enterobactin, along with a specific transport system [5] (Fig. S1). Enterobactin is synthesized in the cytoplasm and exported by the inner and outer membrane transporters EntS and TolC, respectively [6]. Once in the extracellular space, enterobactin chelates iron and forms a complex that interacts with the outer membrane receptor FepA. Transport through FepA towards the periplasm, requires the energy provided by the TonB-ExbB-ExbD system anchored at the inner membrane. Subsequently, the enterobactin-iron complex binds the FepB protein and then interacts with the cytoplasmic pore constituted by FepD and FepG. Next, the ATPase, FepC, facilitates the ferric-enterobactin complex import into the cytoplasm [7]. There, the complex is hydrolyzed by the esterase Fes allowing iron to be released from its coordination with enterobactin hydroxyl groups [8]. Enterobactin is the most avid microbial iron chelator (*Ka* = 10^52^) [8]. Nevertheless, it has been reported that *E. coli* might have up to nine iron transport systems [9]–[17], most of them involving siderophore-iron complexes. In addition, *E. coli* mutants in iron transporters are capable of internalizing iron through the zinc transporter ZupT [18]. Bacteria endowed with multiple iron uptake systems are commonplace in nature [19]. In fact, some bacteria may produce more than one siderophore and also might have the machinery to make use of siderophores produced by other bacteria [19]. This seemingly redundant scenario in terms of iron internalization could indicate that siderophores might have additional and specific functions (*i.e.* in adaptation to different environments). However, until now there is limited experimental evidence enclosing siderophores in alternative physiological roles beyond its known function in iron uptake.

It has been reported that siderophores may be involved in the metabolism of other metals and, specifically, to have a role in heavy metal tolerance [20]–[22]. Furthermore, it was reported that binding of iron-siderophore complexes to specific receptors can trigger signal transduction mechanisms, that affect the expression of genes involved not only in iron uptake [23]. In addition, a number of hydroxamate siderophores including desferrioxamine E were shown to have inhibitory growth activity or to reduce biofilm development in some species [24]–[27]. In line with the alternative role of siderophores, it was demonstrated that desferrioxamine E promotes antibiotic production and has a modulatory effect on the colony development within the genus *Streptomyces* [28]. Additional evidence connecting siderophores and bacterial growth was found through experiments in which “non-cultured” microorganisms became “cultured” in the presence of heterologous siderophores [19]. While some of the mentioned mechanisms might involve iron metabolism, it is likely the participation of other cellular components aside from those committed to covering iron requirements.

In previous work [29] we showed that the Pseudomonad siderophore pyochelin has antibiotic activity against some bacteria. We observed a differential sensitivity to pyochelin in a panel of bacteria tested where catecholate siderophore-producing strains were resistant to pyochelin, while strains producing other type of siderophores were sensitive. Further analysis allowed us to correlate the antibiotic activity of pyochelin with the generation of reactive oxygen species (ROS) and the catechol mediated protection with the ability to reduce ROS [29]. Although *E. coli* has many defense mechanisms against oxidative stress, our results showed that the absence of enterobactin resulted in decreased growth in the presence of a compound that generates ROS [29]. Considering the putative role of enterobactin as an oxidative stress protector, and the association of defective growth phenotypes with oxidative stress (e.g. viable but not culturable state) [30], [31], we decided to analyze the impact of enterobactin absence on *E. coli* growth.

In the present work, we demonstrate that enterobactin plays a critical role in *E. coli* colony development in a minimal medium. We show that instead of the iron uptake impairment, it is the oxidative stress associated with the absence of enterobactin a key element in preventing cells lacking the siderophore from developing colonies. Therefore, we suggest that this siderophore has an oxidative stress protection effect that would be primarily required for colony development.

## Results and Discussion

### The Lack of Enterobactin Affects *E. coli* Growth

**Figure 1:**
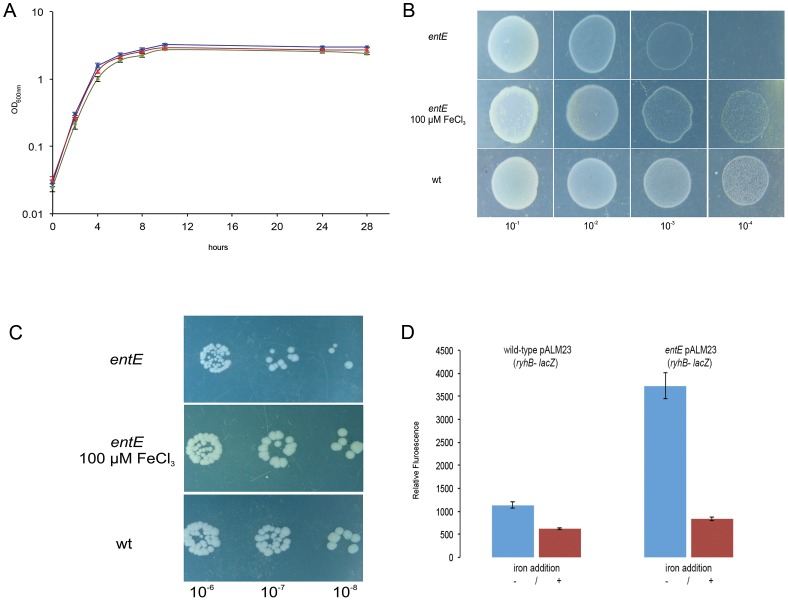
Growth of *E. coli* wild-type (wt) and *entE* strains in liquid and solid media. A) Liquid aerated minimal M9 medium cultures of wild-type strain (blue squares), *entE* strain (green circles) and *entE* strain in the same media but supplemented with 100 µM FeCl_3_ (red triangles). Growth (OD_600_) was determined at the indicated times. B) Lawn growth of wt and *entE E. coli* strains on M9A. A stationary phase culture of *entE E. coli* strain was serially diluted (10^−1^ to 10^−4^) and an aliquot of these dilutions was applied on M9A or M9A supplemented with 100 µM FeCl_3_. As control, the same dilutions of a wt strain overnight culture were applied on M9A medium. Lawn growth was compared at 8 hours of incubation. C) Colony growth of wt and *entE E. coli* on LBA. A stationary phase culture of *entE E. coli* strain was serially diluted and an aliquot of dilutions 10^−6^ to 10^−8^ were applied on LBA or LBA supplemented with 100 µM FeCl_3_. As control, the same dilutions of an overnight culture of the wt strain were applied on LBA medium. After overnight incubation, colonies sizes were compared. D) Activity of the *rhyB* promoter estimated by β-galactosidase activity as an indirect measure of the intracellular iron content (The higher the promoter expression, the lower the iron content [48]). Both wild-type strain and *entE* mutant respond to iron addition. The plasmid pALM23 carries the *ryhB- lacZ* fusion.

**Figure 2:**
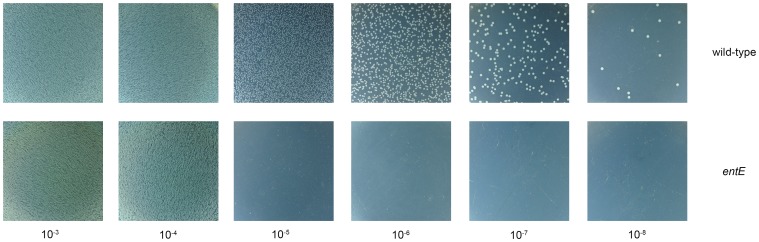
Observed type of growth of *entE* and wild-type strains in M9A after plating and overnight incubation of serial dilutions obtained from stationary phase cultures. Representative pictures show the characteristic leap from lawn growth (10^−4^ dilution) to absence of colonies (10^−5^ dilution) for the *entE* strain in M9A.

We previously showed that a strain impaired in enterobactin biosynthesis had significantly higher levels of reactive oxygen species compared with the wild-type strain when grown in M9 medium [29]. Thus, we assigned a putative physiological role for enterobactin in reducing cell oxidative stress. Then, we wondered if the lack of enterobactin would affect cells growth as a consequence of an imbalanced response to oxidative stress. To analyze this hypothesis, we cultured *entE E. coli* and wild-type *E. coli* in liquid aerated minimal M9 medium. Interestingly, despite of not synthesizing enterobactin, the *entE* strain was able to grow in minimal media, at a comparable rate to that of the wild-type strain (Fig. 1A). The observed growth differences between both strains (Fig. 1A), were not as remarkable as it could be expected, considering the relatively low iron content of M9 medium (1.8 µM) [32] and that enterobactin is the most important iron uptake facilitator that *E. coli* has [7], [33]. In addition, when medium was supplemented with iron (100 µM), growth of the mutant strain increased and reached OD values close to those of the wild-type strain (Fig. 1A). Thus, the differential growth rate observed for the *entE* mutant in liquid aerated culture could be mostly attributed to the iron scavenging property of enterobactin. Nevertheless, when *entE* cells in stationary phase were plated in M9 agar medium (M9A) and incubated overnight for colony count, there was no growth at dilutions where colony formation was expected (Fig. 2). Unlike *E. coli entE*, wild-type *E. coli* formed colonies in the conditions used. Figure 2 shows the type of growth observed for the *entE* mutant and the wild-type strain after respective culture dilutions were plated on M9A and incubated overnight. The inability of *entE* mutant to form colonies in M9A is an interesting growth phenotype since this strain grows normally on LB agar medium (LBA) (Table 1). Table 1 describes the type of growth (lawn or isolated colonies) for the dilutions plated on different media and conditions. For *entE* and wild-type cultures in all conditions used we obtained lawns with 10^−4^ dilutions and below (Table 1). For the wild-type, colonies were observed for 10^−5^ dilutions and above. In contrast, the *entE* mutant showed no growth at these dilutions (Fig. 2 and Table 1). This meant an abrupt growth interruption for *entE* strain at higher dilutions and led us to inquire about the lawn thickness of both wild-type and *entE* strains. To address this question, we estimated the cellular densities of spots obtained after overnight incubation of 10^−4^ dilutions for both strains. This was done by scraping cells off each spot and subsequently counting CFU (both strains had approximately 2×10^9^ cells, Table S1) (see material and methods section). Then, the absence of colony formation in the 10^−5^ dilution plates could not be attributed to the dilution effect and pointed out a defect in colony growth for the *entE* mutant in the culture conditions used. Similar results were observed for dilutions of exponentially growing cultures (data not shown). This indicated that this phenotype is independent from the physiological state of the plated cells. However the abrupt growth arrest at higher dilutions in the case of the *entE* mutant, reveals the necessity of enterobactin particularly at cellular densities that lead to colony formation. The normal growth of *entE* cells on LBA (Table 1) implies that the observed atypical growth on M9A depends on the culture medium composition. Given that iron addition enhanced *entE* growth in aerated M9 liquid cultures (Fig. 1A) and that LB iron content is significantly higher than in M9 medium (LB: 17 µM/M9: 1.8 µM) [32], we hypothesized that iron availability could be the most likely reason for the differential growth. Then, we supplemented M9A medium with an excess of iron (100 µM) and evaluated its effect on the absence of colony formation. Curiously, we observed that iron addition in either, the initial liquid M9 culture, the solid media M9A (where dilutions were plated) or both, had no effect on the colony development arrest (Table 1). In order to assess if the *entE* mutant strain was able to respond to iron supplementation (100 µM FeCl_3_) when growing in agar media, we followed its growth as a lawn on M9A and as colonies on LBA (Fig. 1B and 1C, respectively). We found that, as with the *entE* liquid cultures (Fig. 1A), iron supplementation in M9A made *entE* lawn growth similar to that of the wild-type strain growing without iron addition (Fig. 1B). The observation was made at 8 h of incubation, a time point where lawn growth differences were clearly observed. Therefore, despite that *entE* cells cannot form colonies in M9A, they are able to grow as a lawn and increase this type of growth upon iron addition. Furthermore, in LBA where *entE* is able to form colonies, iron addition increased colony size making it similar to that of the wild-type strain (Fig. 1C). Results indicate that in all culture conditions assayed, iron is available for *entE* cells even though they cannot synthesize enterobactin. Additional evidence of the *entE* strain ability to internalize iron is the repression of the fur regulated promoter *rhyB* (in an *entE* context) to levels comparable to the wild-type strain, upon medium supplementation with 100 µM FeCl_3,_ (Fig. 1D). Taken together, these results suggest that in the absence of enterobactin, other iron uptake systems compensate for the iron requirements. Therefore, enterobactin would play in the conditions used, a critical role in colony development, a process that would not be associated with iron shortage.

**Table 1:** Type of growth of *entE* mutant in solid medium.

			Type of growth for each dilution plated					
Strain	Aerated liquid culture	Solid medium where dilutions were plated	10^−3^	10^−4^	10^−5^	10^−6^	10^−7^	10^−8^
wt	M9	M9A	L	L	C	C	C	C
*entE*	M9	M9A	L	L	NG	NG	NG	NG
*entE*	M9	LBA	L	L	C	C	C	C
*entE*	M9+ Fe^a^	M9A	L	L	NG	NG	NG	NG
*entE*	M9	M9A+ Fe	L	L	NG	NG	NG	NG
*entE*	M9+ Fe	M9A+ Fe	L	L	NG	NG	NG	NG
*entE*	M9	M9A+ ASC^b^	L	L	C	C	C	C
*entE*	M9	M9A+ CAS^c^	L	L	C	C	C	C

One hundred µL of serial dilutions (from 10^−3^ to 10^−8^) from a stationary phase culture were plated in the specified solid medium (M9 agar, M9A or LB agar, LBA) and incubated overnight. The type of growth was observed: L, lawn; C, colony, NG, no growth.

^a^+ Fe, indicate medium supplementation with 100 µM FeCl_3._

b + ASC indicate medium supplementation with 1 mM ascorbic acid.

c +CAS indicate medium supplementation with 1% casamino acid.

### Oxidative Stress in *entE* Cells Leads to the Inability to Form Colonies

**Figure 3:**
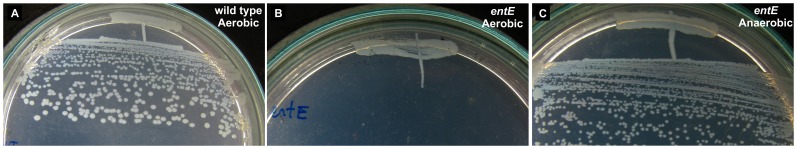
Growth of *E. coli* wild-type and *entE* mutant streaked in M9A. Wild-type strain growth in aerobic conditions (A) and *entE* mutant growth in aerobic (B) or anaerobic conditions (C).

**Figure 4:**
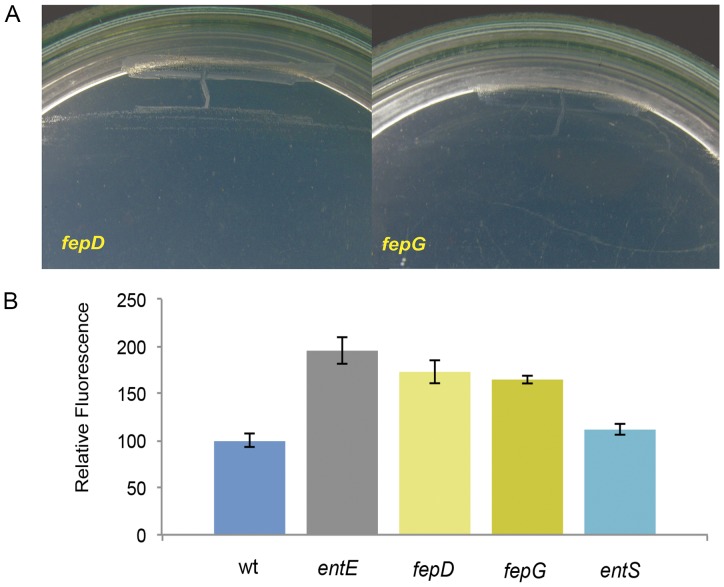
A) Type of growth of *E. coli fepD* and *fepG* mutants streaked in M9A and incubated overnight in aerobic conditions. B) Levels of reactive oxygen species in *E. coli* wild-type and *fepD*, *fepG, entS* and *entE* mutants grown in M9 medium. Quantitation of ROS levels was done using the DCFA-DA probe. Fluorescence intensities are relative to that of the control. Control: wt grown in M9 medium. Error bars = SD, n = 3.

Since we previously reported a putative role for enterobactin in reducing reactive oxygen species [29], we wondered if *entE* mutants’ impairment in colony formation would be connected to oxidative stress. In fact, media supplementation with the reducing agent ascorbic acid allowed *entE* cells to form colonies in M9A (Table 1). Consistent with the observed lawn growth at high cellular densities and no growth at low cellular densities in M9A count plates (Fig. 2), when *entE* strain was streaked in M9A and incubated in aerobic conditions, only lawn growth was observed (Fig. 3B). On the contrary, when it was incubated under anaerobic conditions it was able to form colonies (Fig. 3C) similar to those of the wild-type strain grown aerobically (Fig. 3A). In concordance with these results, we observed that strains able to produce enterobactin but impaired in its internalization into the cytoplasm (*fepD* and *fepG* mutants) displayed the same phenotype in aerobic culture conditions (Fig. 4A). Similarly as reported previously for *entE* mutants, we observed increased ROS levels for *fepD* and *fepG* mutants compared with the wild-type strain (Fig. 4B). This suggests that in order to reduce oxidative stress, enterobactin has to be internalized and reach the cell cytoplasm. Therefore, a mutant defective in enterobactin export should accumulate enterobactin, rendering it unable to use this system to internalize iron, and should be fully functional in terms of ROS scavenging. Thus, we evaluated the colony formation phenotype and the ROS levels of the *entS* mutant. This mutant showed a normal growth phenotype (data not shown) and as expected ROS levels were lower than those of the *entE* strain and similar to levels obtained with the wild-type strain (Fig. 4B). These observations allowed us to assign an oxidative stress protection effect of enterobactin that would occur in the cytoplasm and that would be primarily required for colony development. Results with *entS* mutant decouple the ability of enterobactin to reduce ROS from its fundamental role in iron uptake. However, in physiological conditions where enterobactin is probably exported immediately after being synthesized, enterobactin mediated ROS scavenging would likely require its internalization along with iron.

Since *entE* mutant grew normally at high cellular densities, the ROS protection function of enterobactin would not be as critical for lawn growth (Fig. 2 and Fig. 3B). These results are in concordance with previous reports that link high cellular density with tolerance of microorganisms to adverse conditions such as antibiotics, toxic agents and oxidative stress [34], [35]. In fact, Ma & Eaton [36], demonstrated that the absence of catalase activity rendered cells particularly sensitive to hydrogen peroxide at low cellular densities, therefore implying a counterbalancing effect at high cellular densities. It remains to be evaluated the mechanism by which high cellular density of the *entE* mutant strain overcomes the oxidative stress to allow growth.

### Medium Composition Influence on Oxidative Stress of *entE* Cells

**Figure 5:**
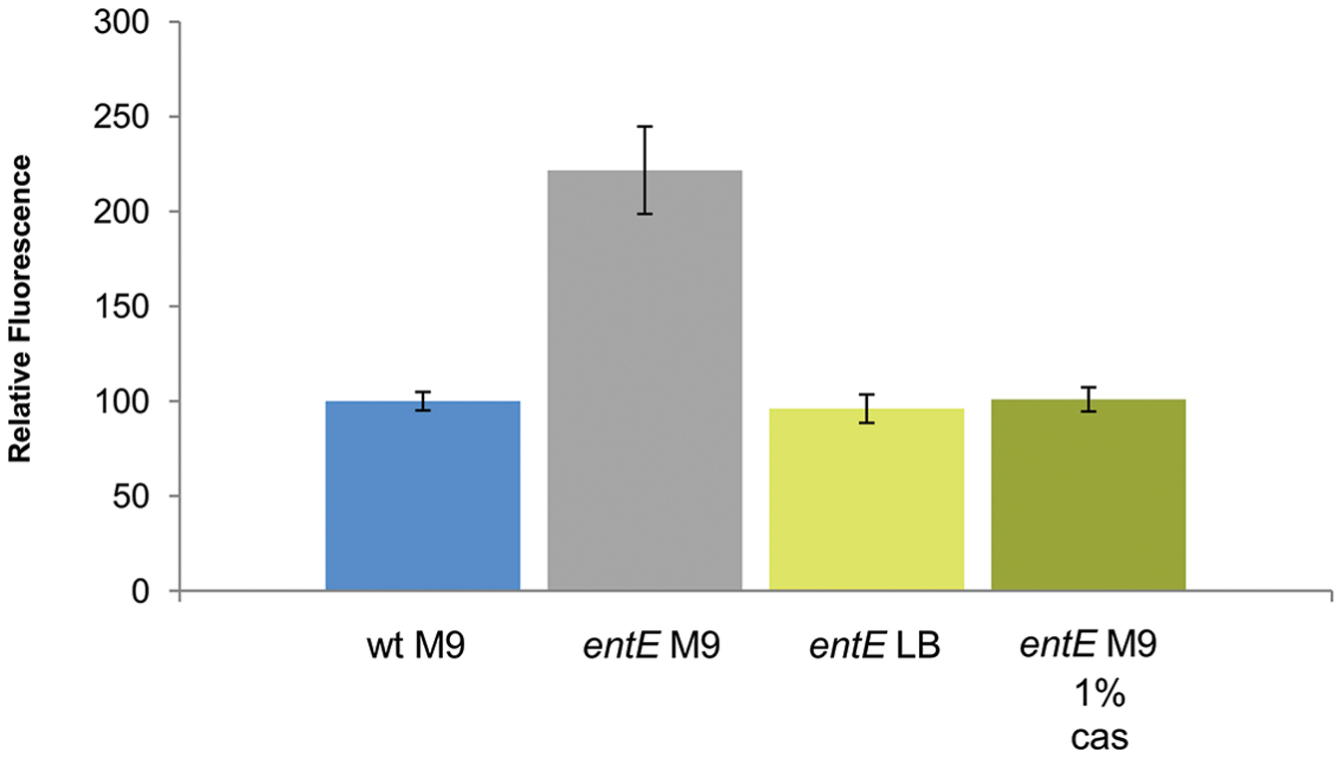
Reactive oxygen species levels in *E. coli entE* mutant grown in different culture media. Quantitation of ROS levels was done using the DCFA-DA probe. Fluorescence intensities are relative to that of the control. Control: wild-type strain grown in M9 medium; *entE* M9: indicates cells grown in M9 medium, *entE* LB: indicates cells grown in LB medium, *entE* M9 1% cas: indicates cells grown in M9 medium supplemented with 1% casamino acids. Error bars = SD, n = 3.

We observed that the *entE* mutant was unable to form colonies when grown in M9A, however normal colonies were obtained in LBA. Then, we analyzed media composition in order to unravel the cause for the differential growth in both media. Since supplementation of LBA with phosphate (40 mM) did not arrest colony formation and since *entE* strain still showed the phenotype in a low phosphate minimum medium (MTA) (data not shown), we ruled out the high phosphate content of M9A as a cause. LBA is a much more complex and nutritious medium than M9A, therefore we hypothesized that higher nutrient availability could favor colony formation in LBA. Therefore, we evaluated the effect of increasing casamino acids content of M9A on colony growth. To our surprise, colony development was restored by increasing casamino acids content from 0.2% up to 1% (Table 1). Then, we decided to analyze whether media composition would correlate with oxidative stress levels. For that, we measured ROS levels for *entE* cells in standing cultures in the same media where colonies developed (LB and M9 plus 1% casamino acids) and in the medium where they did not (M9). Figure 5 shows that ROS levels are increased for *entE* cells grown in M9 compared with *entE* cells in LB or M9 with 1% of casamino acids. In addition, ROS levels for the wild-type strain grown in M9 were similar to those of the *entE* mutant in LB (Fig. 5). These results suggest that lowered ROS levels could be a consequence of the higher amino acid content (in LB and M9 with 1% casamino acids) which is probably reducing ROS through direct radical scavenging [37]. Then, in 0.2% casamino acids M9 medium, enterobactin would play a significant role as an oxidative stress protector that allows colony formation. Accordingly, we observed no colony development in other saline minimal medium (MTA) and normal growth in other rich media (BHI and YEM) (Data not shown).

### Colony Formation Resumes by Reducing Oxidative Stress

**Figure 6:**
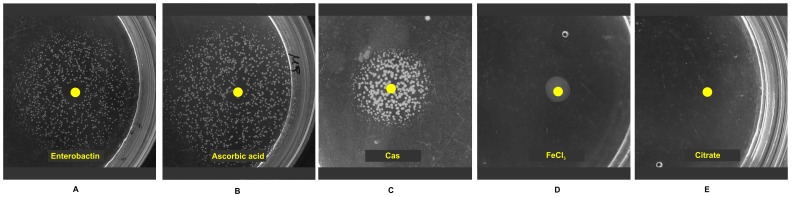
Colonies resume growth upon enterobactin, ascorbic acid or casamino acids (cas) addition. In plates in which no growth was obtained after overnight incubation (10^−5^ dilutions), 1 µL of 1 µM enterobactin (A), 5 µL of 1 mM ascorbic acid (B), 5 µL of 2% casamino acids (C), 10 µL of 1 mM FeCl_3_ (D) or 10 µL of 1 mM sodium citrate (E) were spotted. After a second overnight incubation, a size gradient of colonies was clearly observed around the spots containing enterobactin (A), ascorbic acid (B) and casamino acids (C). However, no growth was observed with FeCl_3_ (D) or citrate (E).

Lawn growth of *entE* mutant and its inability to form colonies in M9A could imply that *entE* cells at low cellular densities enter into a physiological state where colony development is arrested. To address this hypothesis we used the following strategy: in plates in which no growth was obtained after 24 h of incubation (10^−5^ dilutions of an overnight culture) we spotted 1 µL of pure enterobactin (1 µM), reincubated plates and looked for colony formation. Interestingly, we observed a size gradient of colonies around the spot containing enterobactin (Fig. 6A). This physiological complementation indicated us that the absence of colony development is a reversible phenomenon and that enterobactin addition is able to restore colony growth. As was expected, ascorbic acid addition had a similar effect (Fig. 6B) and therefore strengthened the idea that oxidative stress is a key element in arresting colony development. Furthermore, casamino acids were also able to rescue *entE* cells from the arrest state (Fig. 6C) probably by reducing oxidative stress levels as was implied above (Fig. 5). In contrast, neither iron (20 mM) nor citrate (1 mM) addition restored colony formation (Fig. 6D–E). This is relevant since by expressing its cognate membrane receptor FecA, *E. coli* can use citrate as bona fide siderophore [38]. Besides, citrate-iron complexes can further augment iron uptake through the iron starvation sigma factor FecI, which increases the expression of genes involved in iron metabolism [39]. Therefore, these results support the idea that iron availability is not accountable for the impaired colony development of *entE* cells. Finally, since both citrate and enterobactin facilitate iron uptake, there must be a structural trait of enterobactin that is responsible for lessening the oxidative stress and therefore allows colony formation.

### Enterobactin Hydrolysis would be Required for Oxidative Stress Protection

**Figure 7:**
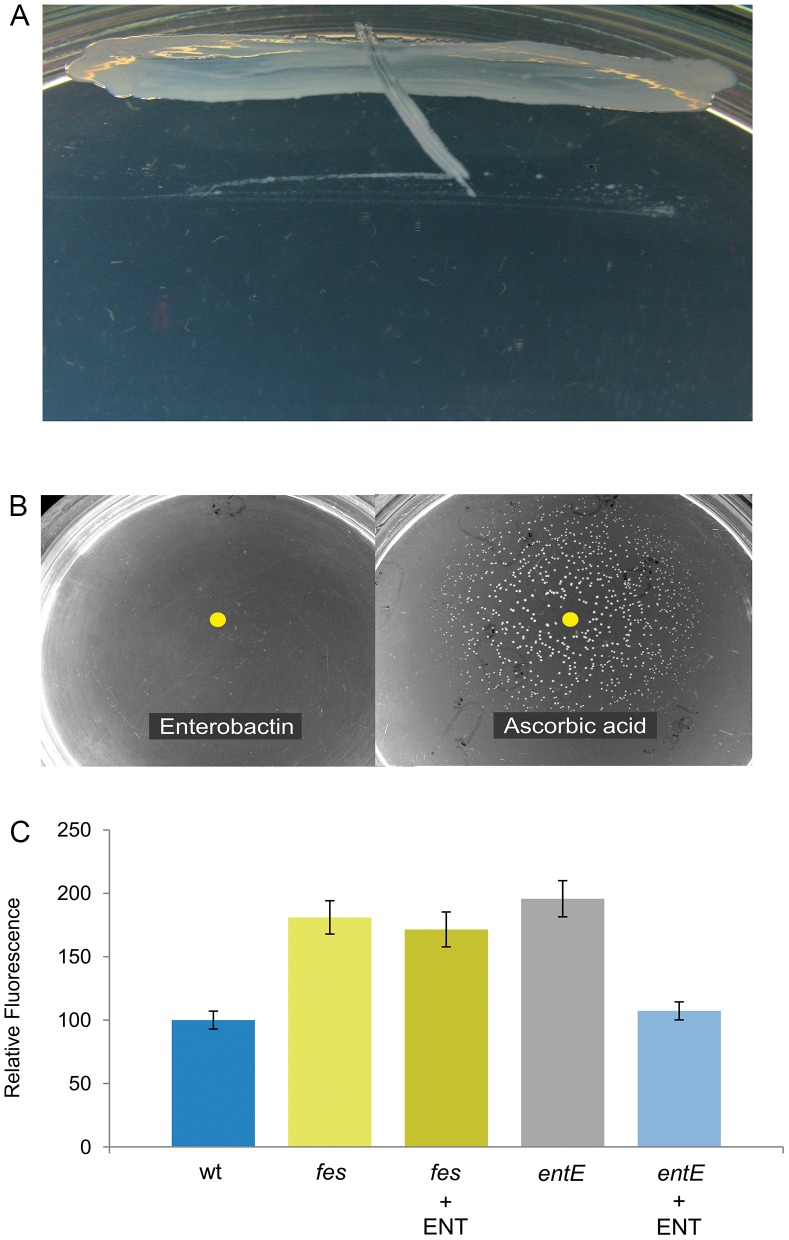
A) Type of growth of *E. coli fes* mutant streaked in M9A and incubated overnight in aerobic conditions. B) No colony development was observed when a 10^−5^ dilution of a *fes* mutant stationary phase culture was incubated overnight. Then, 1 µL of pure enterobactin (1 µM) or 5 µL ascorbic acid (1 mM) were spotted on the medium surface and reincubated overnight. It can be observed that ascorbic acid restores colony growth of *fes* mutant meanwhile enterobactin does not. C) Reactive oxygen species levels in *E. coli fes* and *entE* mutants grown in M9 medium. Quantitation of ROS levels using the DCFA-DA probe. Fluorescence intensities are relative to that of the control. Control: wt strain grown in M9 medium; +ENT: indicates addition of 1 µM enterobactin. Error bars = SD, n = 3.

Enterobactin structure has a central triserine macrocycle linked to three lateral catecholate moieties which are involved in iron complexation [7]. Due to their low radical reduction potentials, catechols can act as hydrogen atom donors and efficiently terminate radical chain reactions [40]. This ability requires hydroxyl moieties to be available for radical scavenging [41]. Accordingly, we observed that a mutant unable to free iron from the enterobactin-iron complex (*fes*) did not form colonies in M9A (Fig. 7A). Furthermore, meanwhile enterobactin addition did not resume *fes* strain colony development, ascorbic acid did (Fig. 7B). As expected, the *fes* mutant showed increased ROS levels compared with the wild-type strain and enterobactin supplementation was not able to lower ROS levels, as was previously observed for the *entE* strain (Fig. 7C) [29]. These results indicate that enterobactin not only has to reach the cell cytoplasm (Fig. 4A) but also has to be hydrolyzed in order to allow colony development through oxidative stress protection. Since iron availability does not seem to be involved in the colony growth arrest, we hypothesize that freed hydroxyl groups in the enterobactin molecule are responsible for the oxidative stress protection. Several reports link catechol siderophores with oxidative stress. For example, *Bacillus anthracis* catecholate siderophores, bacillbactin and petrobactin, are overproduced when cells are exposed to oxidative stress [42]. In *Azotobacter vinelandii* it was demonstrated that oxidative stress increased a catecholate siderophore synthesis and that its production was under the control of SoxS [43]. We previously showed that enterobactin protects against the ROS-mediated toxic effects of pyochelin and that impairment of enterobactin biosynthesis resulted in increased ROS levels [29]. Recently, Achard *et al* [44] demonstrated that the catecholate siderophores salmochelin and enterobactin protect *S. Typhimurium* against ROS, specially at early stages of macrophage invasion which correlate with the oxidative burst. Authors also showed that non-catecholate siderophores do not exert this protection and also that catechols need to be internalized in order to fully protect *Salmonella* against the oxidative stress [44].

Therefore, it is conceivable that catechols may be employed as protectants against oxidative stress. *E. coli* has many mechanisms that help cells to cope with oxidative stress. Examples are those coordinated through the OxyR and SoxSR regulons [45]. However, we observed that *oxyR* and *soxS* mutants displayed a normal colony growth in conditions where the *entE* mutant failed to form colonies (M9A) (data not shown). Thus, we hypothesize that enterobactin would be mostly relevant in reducing oxidative stress in situations associated with low cellular densities (e.g. single cells plated to obtain isolated colonies). It is interesting to note that enterobactin hydroxyl moieties would require to be released from the coordination complex with iron, in order to protect from the oxidative stress. Then, this iron uptake system would ensure *E. coli* not only iron entry into the cell, which might lead to oxidative stress through Fenton reaction, but the simultaneous generation of a molecule that scavenges ROS and therefore counterbalances the potential damage that iron excess might cause.

## Materials and Methods

### Bacterial Strains and Growth Conditions

List of strains and plasmids used in this work are showing in Table 2.

**Table 2:** List of strains and plasmids used in this work.

Strains	Relevant genotype	Source
*Escherichia coli* BW 25113	wild type	CGSC^a^
*Escherichia coli* JW 0586-1	BW25113 Δ*entE*::*kan*	CGSC^a^
*Escherichia coli* JW0582-2	BW25113 Δ*fepD*::*kan*	CGSC^a^
*Escherichia coli* JW0581-3	BW25113 Δ*fepG*::*kan*	CGSC^a^
*Escherichia coli* JW0576-2	BW25113 Δ*fes*::*kan*	CGSC^a^
*Escherichia coli* JW 0583	BW25113 Δ*entS*::*kan*	Bernhardt lab
*Escherichia coli* JW3933-3	BW25113 Δ*oxyR::kan*	CGSC^a^
*Escherichia coli* JW4023-5	BW25113 Δ*soxS::kan*	CGSC^a^
**Plasmids**		
pALM23	*ryhB-lacZ* transcriptional fusion in pQ50	Ma L & Payne SM [48]

a CGSC, *Escherichia coli* Genetic Stock Center;

Strains were grown in either LB (Sigma-Aldrich) and M9 (Sigma-Aldrich) medium supplemented with 0.2% or 1% casamino acids, 0.2% glucose, 1 mM MgSO_4_ and 1 mg/mL vitamin B1. Solid media contained 1.5% agar. For growth curves, culture aliquots were taken at different times and OD at 600 _nm_ was measured. For the observation of the colony arrest phenotype, 100 µL of serial dilutions (made with M9 medium) from an overnight culture of either *entE* or wild-type strains were plated on the corresponding agar medium and incubated overnight. When indicated, ascorbic acid and FeCl_3_ were supplemented at a final concentration of 1 mM and 100 µM, respectively. Anaerobic cultures were performed in an anaerobic jar containing anaerobic-generating salts (Anaerocult- Merck Millipore). For the colony arrest reversion assay, 1 µL of 1 µM enterobactin (EMC micro collections), 5 µL of 1 mM ascorbic acid (Sigma- Aldrich), 5 µL of 2% casamino acids (Sigma- Aldrich), 10 µL of 1 mM FeCl_3_ (Sigma- Aldrich) or 10 µL of 1 mM sodium citrate (Sigma- Aldrich) were spotted on the surface of plates where no colonies were obtained after overnight incubation (10^−5^ dilution from *entE* overnight culture). To compare the cell density of lawns obtained with 10^−4^ dilutions of *entE* and wild-type overnight cultures, we used the following approach: Four 50 µL fractions of a 10^−4^ dilution from each culture strain (*entE* and wild-type) were spotted onto M9A. After 24 h of incubation each spot was scraped off and resuspended in 1 mL of M9 medium. Serial dilutions were made and CFU count was determined in LBA for quadrupled.

### Measurement of Reactive Oxygen Species

To determine the level of reactive oxygen species (ROS), we used the oxidation-sensitive fluorescent dye 2′, 7′-dichlorodihydrofluorescein diacetate (DCFH-DA). This fluorescent probe is frequently used for detection of reactive oxygen species such as hydrogen peroxide, hydroxyl radical, peroxyl radicals and nitrogen radicals [46]. DCFH-DA is deacetylated by cellular esterases and then converted by reactive species into dichlorofluorescein (DCF), which can easily be visualized by strong fluorescence at 530 nm when excited at 485 nm. Exponentially growing cells in M9 minimal medium, were washed and resuspended in 50 mM sodium phosphate buffer, pH 7 at a final OD_600 nm_ = 0.5. Then DCFH-DA was added at a final concentration of 10 mM and incubated for 30 min [28]. After incubation, cells were washed, resuspended and sonicated in the same buffer. Fluorescence intensity was measured using a Perkin Elmer LS55 spectrofluorometer (excitation λ, 490 nm; emission λ, 519 nm). Results are expressed as relative fluorescence to that of the control.

### β-Galactosidase Assays

The β-galactosidase activity was determined following the method described by Zhou *et al* (Zhou & Gottesman, 1998 [47]). Strains carrying the *ryhB-lacZ* transcriptional fusion were grown in M9 medium at 37°C up to OD_600 nm_ = 0.1, at this point the culture was divided in half and one sample was supplemented with 100 µM FeCl_3_. When cultures reached an OD_600 nm_ = 0.6, the β-galactosidase activities were assayed. For this, 600 µL aliquots of these cultures were permeabilized for 10 min with 0.1% SDS (24 µL) and chloroform (48 µL). Then, 100 µL of permeabilized cells were put onto 96 wells microtiter plate, 100 µL of a 1.32 mg/mL solution of o-Nitrophenyl-β-D-galactopyranoside in buffer Z were added and absorbances at 420 nm were measured for 20 min, in a SpectraMax 250 spectrophotometer. The β-galactosidase activity was calculated by dividing the slope of the line over time by the corresponding OD_600 nm_.

## Supporting Information

Figure S1 **Scheme of enterobactin iron uptake system.**
(TIF)

Table S1 **Comparison of cell density of spots obtained with 10^−4^ dilutions.**
(DOCX)
