# Supplementary figures and images for: The Alternative Role of Enterobactin as an Oxidative Stress Protector Allows Escherichia coli Colony Development
Source: PLoS One. 2014 Jan 2;9(1):e84734. doi: 10.1371/journal.pone.0084734 (PMC3879343; doi:10.1371/journal.pone.0084734)

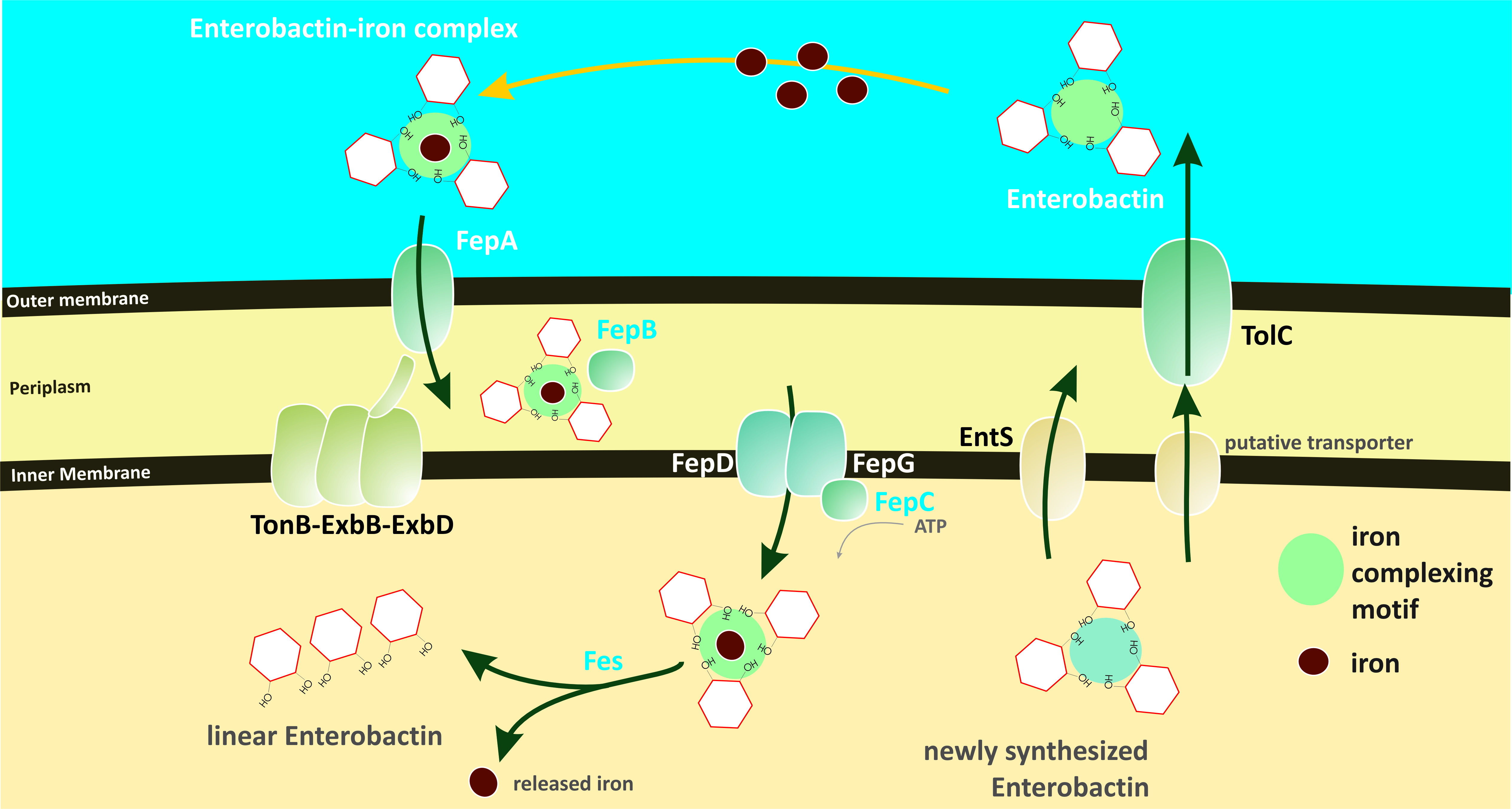

Supplement: Figure S1 — Scheme of enterobactin iron uptake system. (TIF) [file pone.0084734.s001.tif]
