# Supplementary material for: The Alternative Role of Enterobactin as an Oxidative Stress Protector Allows Escherichia coli Colony Development
Source: PLoS One. 2014 Jan 2;9(1):e84734. doi: 10.1371/journal.pone.0084734 (PMC3879343; doi:10.1371/journal.pone.0084734)
Supplement: Table S1 — Comparison of cell density of spots obtained with 10−4 dilutions. (DOCX) [file pone.0084734.s002.docx]

Table S1

Comparison of cell density of spots obtained with 10^- 4^ dilutions

| **Strain** | **CFU count (media ± SD)** |
| --- | --- |
| wild-type | 2,025 x10^9^ ± 0,5 |
| *entE* | 2,125 x10^9^ ± 0,7 |
